# An analytical approach for three-dimensional thermal analysis of moving heat source on orthotropic solid

**DOI:** 10.1177/08927057251344553

**Published:** 2025-05-28

**Authors:** Mahmoud Fereidouni, Suong Van Hoa

**Affiliations:** 1 Concordia Center for Composites, Department of Mechanical, Industrial and Aerospace Engineering, 5618 Concordia University, Montreal, QC, Canada; 2 Research Center for High-Performance Polymer and Composite System (CREPEC), Montreal, QC, Canada

**Keywords:** Analytical solution, moving heat source, orthotropic solid, three-dimensional thermal analysis

## Abstract

The Rosenthal steady-state analytical solution for the temperature distribution caused by a moving point heat source on a semi-infinite, homogeneous, *isotropic* solid has been extensively used in modeling metallurgical processes, e.g., arc welding. This study develops a three-dimensional analytical closed-form solution for the temperature field induced by a point heat source moving across a semi-infinite *orthotropic* solid. The formulation accommodates arbitrary orientations of the heat source’s motion relative to the material’s in-plane principal axes and is extended to solids with finite thickness. Subsequently, using the superposition of the linear solutions, a general methodology is proposed to predict the temperature distribution resulting from an arbitrarily distributed heat source. Verification of the closed-form solution and validation of the distributed heating condition against finite element simulations demonstrate excellent agreement. The analytical framework offers potential for thermal modeling of processing methods used for polymeric composites, e.g., additive manufacturing and continuous welding of thermoplastic composites.

## Introduction

With the widespread integration of automation in the industrial processing of various materials and the manufacturing of structural components, the theoretical study of moving heat sources in localized processing has garnered attention since the 1940s with the advent of automated metal welding, and has remained highly relevant with recent advancements in additive manufacturing processes for metallic alloys. Understanding heat propagation within a material subjected to a moving heat source is critical for predicting its thermal history, which directly governs the evolution of microstructural properties and impacts the final product’s quality in terms of mechanical performance and dimensional stability.

**Figure 1.**
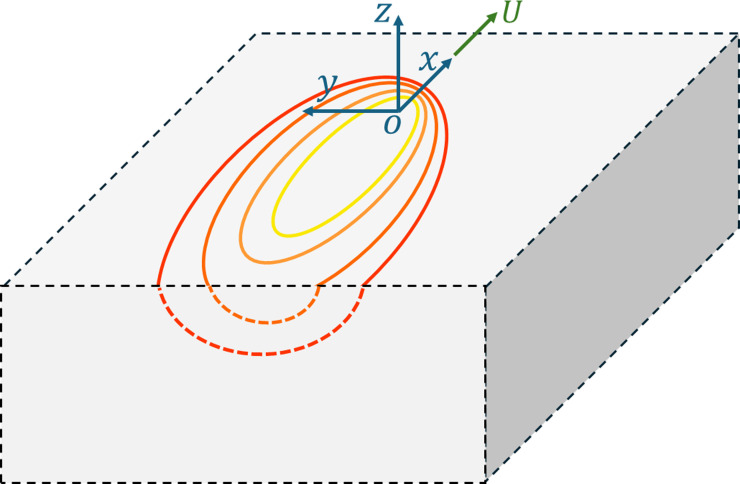
Representation of the thermal field via moving reference frame attached to a moving point heat source on semi-infinite isotropic solid^5^.

Rosenthal^1^ developed a foundational theory for thermal analysis of a moving point heat source on isotropic solids. He provided analytical quasi-steady solutions for one-dimensional, two-dimensional, and three-dimensional heat flow in infinite and semi-infinite solids bounded by planes. Meanwhile, Jaeger^2^ and Carslaw and Jaeger^3^ made seminal contributions in the development of the analytical solutions of the heat conduction equation for different moving source shapes (e.g., band, square, and rectangular with uniform distribution). Later, Hou and Komanduri^4^ extended the solutions for additional shapes (e.g., elliptical and circular geometries) and other heat distributions (e.g., parabolic and normal profiles). Despite the advancements, the original Jaeger-Rosenthal closed-form analytical solution for a moving point heat source has remained the well-established framework in the process modelling of joining or additive manufacturing (e.g., beam welding, arc welding, selective laser melting, and laser metal deposition) of isotropic metallic materials (e.g., stainless steel, aluminum alloys, titanium alloys),^5^ expressing the temperature distribution in a semi-infinite solid as:

(1) $$T=T_{\infty }+\frac{Q^{\prime }}{2\pi kr}e^{-\frac{U}{2\gamma }\left(r+x\right)},$$

where $T_{\infty }$ is the temperature at an infinite distance from the source, $Q^{\prime }$ is the heat power transferred to the semi-infinite solid, k is thermal conductivity, γ is thermal diffusivity, U is magnitude of the source velocity, r is the absolute distance from the source, and x is the distance from the source projected along the velocity vector, as depicted in Figure 1.

**Figure 2.**
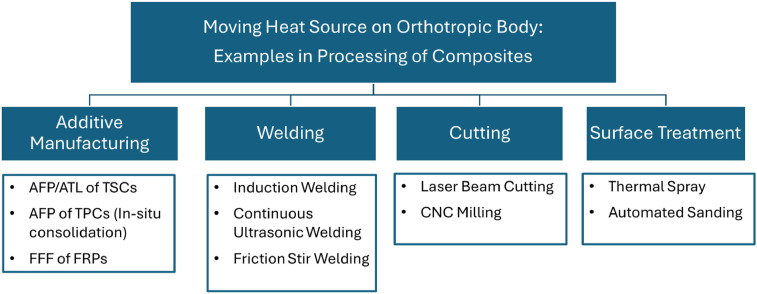
Application of moving heat source in different processes of polymeric composites.

While analytical modeling of a moving heat source on isotropic materials is well-established and no longer considered novel, there remains a need for closed-form analytical solutions for arbitrarily oriented moving heat sources on orthotropic materials, where the thermal conductivity is orientation-dependent. Developing a theoretical framework for orthotropic materials would assist in modeling the thermal processing of fiber-reinforced composites, with examples of polymer-based composites listed in Figure 2. In automated fiber placement (AFP) or automated tape laying (ATL) of thermoset composites (TSCs), typically, an infrared heating system integrated into the placement head increases the temperature of the substrate for enhanced tackiness as the head moves forward during deposition. In AFP of thermoplastic composites (TPCs), where diode laser,^6^ hot gas torch,^7^ or flash lamp^8^ heating systems are typically used to heat the material above its melting point for in-situ consolidation, or for fused filament fabrication (FFF) of fiber reinforced polymers (FRPs) as a similar example, the temperature distribution around the deposition area controls the quality of the final part. A lack of precise prediction over temporal and spatial temperature distribution within the material can lead to violating the process window and causing excessive defects such as material degradation, incomplete healing, and increased void content,^9^ which ultimately compromise the mechanical performance of the structure.^10,11^ Robust and rapid predictive thermal tools allow for online fine-tuning of process parameters, such as the speed, intensity, or distribution of the moving heat source, to achieve the optimal material properties while minimizing energy usage and maximizing efficiency.

Among the original studies on thermal modeling for in-situ consolidation of thermoplastic composites, Tierney and Gillespie^12^ employed a one-dimensional steady-state model to predict the through-thickness temperature distribution when the composite surface is exposed to hot gas impingement. Ghasemi Nejhad et al.^13^ developed a two-dimensional analytical model for the thermal analysis of thermoplastic tape laying, modeling a moving uniform heat source over a transversely isotropic domain. Pitchumani *et al.*^14^ also utilized a two-dimensional thermal model to predict the evolution of interfacial bonding, polymer degradation, and void consolidation, with the aim of defining the optimal processing window. While one- and two-dimensional analytical and numerical models continue to be efficient and popular,^15^ their accuracy might be limited – for instance, by neglecting heat diffusion across the width. This has motivated the adoption of three-dimensional numerical approaches, especially finite element methods,^16^ enabling more accurate thermal predictions, though at the cost of substantially increased computational effort.

The requirement for accurate thermal models is equally critical in continuous in-situ joining techniques for thermoplastic composites (e.g., induction welding,^17^ ultrasonic welding,^18^ friction stir welding^19^) where localized melting and solidification of interfacial thermoplastics occurs within the order of a second during the forward motion of the end-effector. On the other hand, in cutting or surface treatment of composites,^20^ predicting temperature distribution caused by direct heat source (e.g., laser beam, thermal spray, respectively) or friction-induced heat generation (e.g., CNC milling, automated sanding, respectively) may allow for prevention of thermally softened or minimization of degraded material by optimizing process parameters.

The application of an analytical framework for orthotropic solids can even be extended to additively manufactured isotropic materials if significant porosity, characterized by longitudinal voids/gaps along the deposition axis, renders the homogenized properties effectively orthotropic. Beyond polymers and composites, the framework may be relevant to specific metallic substrates exhibiting orthotropic thermal properties. An example could be single-crystal alloys, such as those used in turbine blades,^21^ which display direction-dependent thermal conduction and are subjected to laser-based repair techniques involving localized, moving heat sources.

In this study, an analytical closed-form solution is developed for the temperature distribution induced by a point heat source moving over a semi-infinite orthotropic solid, considering an arbitrary orientation of motion relative to the material’s principal axes. The solution is then extended to accommodate solids with finite thickness. Finally, leveraging the principle of superposition for linear systems, a methodology is proposed to predict the temperature distribution resulting from an arbitrarily distributed heat source. The proposed solutions for the point heat source (in both semi-infinite and finite-thickness cases) and the results for the distributed heat source are validated against finite element simulations, demonstrating excellent agreement.

### Theoretical modelling

The following equation represents the three-dimensional heat conduction for an orthotropic solid in Cartesian coordinates:

(2) $$k_{x}\frac{\partial^{2}T}{\partial {x^{\prime }}^{2}}+k_{y}\frac{\partial^{2}T}{\partial {y^{\prime }}^{2}}+k_{z}\frac{\partial^{2}T}{\partial {z^{\prime }}^{2}}+q=\rho c_{p}\frac{\partial T}{\partial t}\;.$$

The spatial coordinates $x^{\prime }$ - $y^{\prime }$ - $z^{\prime }$ are defined with respect to a stationary reference frame fixed to the material and aligned along its principal axes. Temperature ( T ) can be a function of spatial coordinates and time, while the principal thermal conductivities ( $k_{x},k_{y},k_{z}$ ), specific heat capacity ( $c_{p}$ ), and density ( ρ ) are considered constant properties of the homogeneous domain. The term q represents the rate of internal heat generation per unit volume. In the case of an infinitesimal point heat source, q is nonzero only at the precise location of the heat source and is equal to zero elsewhere throughout the domain. Hence, for a single point heat source located at ( $x_{0}^{\prime },y_{0}^{\prime }$ , $z_{0}^{\prime }$ ) at time t , q can be represented as:

(3) $$q=Q\left(t\right)\delta \left(x^{\prime }-x_{0}^{\prime }\right)\delta \left(y^{\prime }-y_{0}^{\prime }\right)\delta \left(z^{\prime }-z_{0}^{\prime }\right),$$

where Q denotes instantaneous thermal power injected into the system by the point heat source. For now, since the interest is to analyze the heat conduction behavior in the domain excluding the singular point of the heat source, it is evident that q=0 .

**Figure 3.**
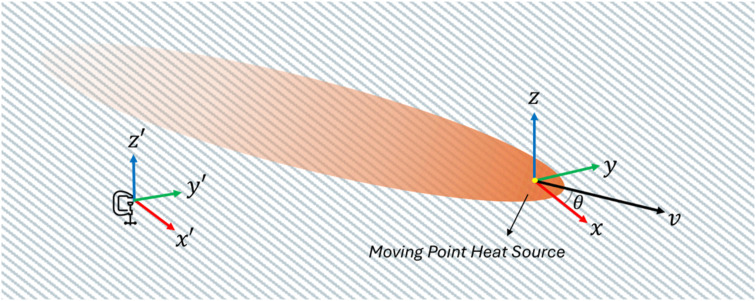
Representation of stationary $x^{\prime }$ - $y^{\prime }$ - $z^{\prime }$ reference frame, and moving x - y - z reference frame attached to the moving point heat source inside the infinite orthotropic solid (x-axis oriented along the principal axis of the material, e.g., fibers).

Consider a point heat source moving horizontally ( $v_{z}=0$ ) inside an infinite solid domain at a constant velocity v . It will be shown that this particular choice reflects the configuration of the most relevant applications – such as additive manufacturing and continuous welding – where the end-effector traverses a horizontal path. A moving reference frame is defined such that it is fixed to the point heat source but remains aligned with the principal axes of the orthotropic material, resulting in a coordinate transformation to the x - y - z system, as illustrated in Figure 3.

Let θ denote the angle between the velocity vector *v* and the positive x-axis. Accordingly, the components of the velocity vector are $v_{x}=v\;\cos\left(\theta \right)$ and $v_{y}=v\;\sin\left(\theta \right)$ . Now, the relationship between the stationary and moving coordinate systems is described by:

(4) $$x=x^{\prime }-v_{x}t,$$

(5) $$y=y^{\prime }-v_{y}t,$$

(6) $$z=z^{\prime }\;.$$

To mathematically simplify the orthotropic nature, spatial coordinates and velocity components can be transformed into scaled variables so that they are normalized based on the corresponding thermal conductivities:

(7) $$X=\frac{x}{\sqrt{k_{x}}},$$

(8) $$Y=\frac{y}{\sqrt{k_{y}}},$$

(9) $$Z=\frac{z}{\sqrt{k_{z}}},$$

(10) $$X^{\prime }=\frac{x^{\prime }}{\sqrt{k_{x}}},$$

(11) $$Y^{\prime }=\frac{y^{\prime }}{\sqrt{k_{y}}},$$

(12) $$Z^{\prime }=\frac{z^{\prime }}{\sqrt{k_{z}}},$$

(13) $$V_{x}=\frac{v_{x}}{\sqrt{k_{x}}},$$

(14) $$V_{y}=\frac{v_{y}}{\sqrt{k_{y}}}\;.$$

With these, equations (4), (5), and (6) transform to:

(15) $$X=X^{\prime }-V_{x}t,$$

(16) $$Y=Y^{\prime }-V_{y}t,$$

(17) $$Z=Z^{\prime }\;.$$

By the transformation of partial derivatives using the chain rule, the quasi-steady heat equation can be expressed in the new coordinates as follows:

(18) $$\frac{\partial^{2}T}{\partial X^{2}}+\frac{\partial^{2}T}{\partial Y^{2}}+\frac{\partial^{2}T}{\partial Z^{2}}=-\rho c_{p}V_{x}\frac{\partial T}{\partial X}-\rho c_{p}V_{y}\frac{\partial T}{\partial Y}\;.$$

Inspired by Rosenthal,^1^ to mathematically separate the effects of advection and symmetric heat diffusion, and to further simplify equation (18), consider:

(19) $$T-T_{\infty }=e^{-\frac{1}{2}\rho c_{p}V_{x}X}e^{-\frac{1}{2}\rho c_{p}V_{y}Y}\varphi \left(X,Y,Z\right),$$

where $T_{\infty }$ represents the temperature of the solid far from the influence of the heat source, where temperature gradients are effectively zero. The first and second terms on the right-hand side describe the exponential decay in the positive *X* and *Y* directions, respectively, as a direct result of the heat source moving forward. The function φ(X,Y,Z) , which accounts for the effective symmetric heat diffusion, must be determined to satisfy equation (18). By substituting equation (19) into equation (18) and applying the necessary manipulations (detailed derivations are shown in Appendix A):

(20) $$\frac{\partial^{2}\varphi }{\partial X^{2}}+\frac{\partial^{2}\varphi }{\partial Y^{2}}+\frac{\partial^{2}\varphi }{\partial Z^{2}}-{\left(\frac{1}{2}\rho c_{p}\sqrt{{V_{x}}^{2}+{V_{y}}^{2}}\right)}^{2}\varphi =0\;.$$

This equation appears symmetric with respect to the coordinates, and technically, φ is invariant of orientation. In this case, let us define a new parameter:

(21) $$s=\sqrt{X^{2}+Y^{2}+Z^{2}},$$

where s accounts for the radius of a sphere in the X - Y - Z coordinates (or an ellipsoid in the x - y - z coordinates). Meanwhile, the Laplacian in spherical coordinates ( s - α - β ) appears as:

(22) $$\nabla^{2}=\frac{1}{s^{2}}\frac{\partial }{\partial s}\left(s^{2}\frac{\partial }{\partial s}\right)+\frac{1}{s^{2}\;\sin\alpha }\frac{\partial }{\partial \alpha }\left(\sin\alpha \frac{\partial }{\partial \alpha }\right)+\frac{1}{s^{2}\;\sin^{2}\alpha }\frac{\partial^{2}}{\partial \beta^{2}}\;.$$

Since φ is radially symmetric ( φ=φ(s) ), the derivatives with respect to α and β vanish. Therefore, by transforming the Laplacian from Cartesian to spherical coordinates, the equation (20) transforms into:

(23) $$\frac{1}{s^{2}}\frac{\partial }{\partial s}\left(s^{2}\frac{\partial \varphi }{\partial s}\right)-{\left(\frac{1}{2}\rho c_{p}\sqrt{{V_{x}}^{2}+{V_{y}}^{2}}\right)}^{2}\varphi =0\;.$$

Let us define g(s)=sφ(s) . Then, equation (23) becomes:

(24) $$\frac{\partial^{2}g}{\partial s^{2}}-{\left(\frac{1}{2}\rho c_{p}\sqrt{{V_{x}}^{2}+{V_{y}}^{2}}\right)}^{2}g=0\;.$$

The solution of this linear second-order homogeneous differential equation is:

(25) $$g=C_{1}e^{-\frac{1}{2}\rho c_{p}\sqrt{{V_{x}}^{2}+{V_{y}}^{2}}\;s}+C_{2}e^{+\frac{1}{2}\rho c_{p}\sqrt{{V_{x}}^{2}+{V_{y}}^{2}}\;s},$$

or

(26) $$\varphi =\frac{C_{1}}{s}e^{-\frac{1}{2}\rho c_{p}\sqrt{{V_{x}}^{2}+{V_{y}}^{2}}\;s}+\frac{C_{2}}{s}e^{+\frac{1}{2}\rho c_{p}\sqrt{{V_{x}}^{2}+{V_{y}}^{2}}\;s}\;.$$

However, the physics of the problem requires $C_{2}$ to be zero, since the influence of the heat source must decay to zero at infinity. By applying this and substituting equation (26) into equation (19):

(27) $$T=T_{\infty }+e^{-\frac{1}{2}\rho c_{p}\left(V_{x}X+V_{y}Y\right)}\frac{C_{1}}{s}e^{-\frac{1}{2}\rho c_{p}\sqrt{{V_{x}}^{2}+{V_{y}}^{2}}\;s}\;.$$

Up to this point, the boundary condition at infinity has been applied, leading to $C_{2}=0$ . To determine $C_{1}$ , it is necessary to apply the remaining boundary condition, which is the singular point of the heat source where the heat power Q is injected. This requires incorporating the term q back into the analysis, as the singular point of the heat source is now of primary interest. By applying a transformation to equation (3), converting from the $x^{\prime }$ - $y^{\prime }$ - $z^{\prime }$ coordinates to the X - Y - Z coordinates:

(28) $$q=\frac{Q}{\sqrt{k_{x}}\sqrt{k_{y}}\sqrt{k_{z}}}\;\delta \left(X\right)\delta \left(Y\right)\delta \left(Z\right)\;.$$

To simplify the determination of the constant $C_{1}$ , a special case is considered where the velocity of the point heat source is zero. Under this condition, firstly, equation (27) simplifies to:

(29) $$T=T_{\infty }+\frac{C_{1}}{s}\;.$$

Secondly, the heat equation in the transformed coordinates may appear as:

(30) $$\frac{\partial^{2}T}{\partial X^{2}}+\frac{\partial^{2}T}{\partial Y^{2}}+\frac{\partial^{2}T}{\partial Z^{2}}=-\frac{Q}{\sqrt{k_{x}}\sqrt{k_{y}}\sqrt{k_{z}}}\;\delta \left(X\right)\delta \left(Y\right)\delta \left(Z\right)\;.$$

Let us define $P=\frac{Q}{\sqrt{k_{x}}\sqrt{k_{y}}\sqrt{k_{z}}}$ , assigning it artificial dimension of [W]. With this definition, equation (30) resembles heat propagation from a point source with heat power P in an isotropic solid, assuming a thermal conductivity of k=1 [W/m⋅K] in the X - Y - Z coordinate system. Also, based on Fourier’s law of heat conduction in the radial direction:

(31) $${\bar{q}}_{s}=-k\frac{\partial T}{\partial s}\;.$$

Here, ${\bar{q}}_{s}$ denotes the rate of heat conduction per unit area in the radial direction. In the particular scenario of heat propagation from a point source within an infinite isotropic solid (i.e., purely spherical diffusion), the radial heat flux corresponds to the input heat power divided by the area of a spherical surface centered at the point of heat injection. Based on this, and considering equation (29), equation (31) can be reformulated as:

(32) $$\frac{P}{4\pi s^{2}}=\frac{C_{1}}{s^{2}}\;.$$

Therefore:

(33) $$C_{1}=\frac{Q}{4\pi \sqrt{k_{x}}\sqrt{k_{y}}\sqrt{k_{z}}}\;.$$

With the constant $C_{1}$ determined, the general form of equation (27) becomes:

(34) $$T=T_{\infty }+\frac{Q}{4\pi \sqrt{k_{x}k_{y}k_{z}}\;s}e^{-\frac{1}{2}\rho c_{p}\left(V_{x}X+V_{y}Y+\sqrt{{V_{x}}^{2}+{V_{y}}^{2}}\;s\right)}\;.$$

By substituting equations (7), (8), (9), (13), (14), and (21) into equation (34), the temperature distribution in the x - y - z coordinate system can be expressed as:

(35) $$T=T_{\infty }+\frac{Q}{4\pi \sqrt{k_{x}k_{y}k_{z}}\;\sqrt{\frac{x^{2}}{k_{x}}+\frac{y^{2}}{k_{y}}+\frac{z^{2}}{k_{z}}}}{\;e}^{-\frac{1}{2}\rho c_{p}v\left(\frac{x\;\cos\;\theta }{k_{x}}+\frac{y\;\sin\;\theta }{k_{y}}+\sqrt{\frac{\cos^{2}\;\theta }{k_{x}}+\frac{\sin^{2}\;\theta }{k_{y}}}\;\sqrt{\frac{x^{2}}{k_{x}}+\frac{y^{2}}{k_{y}}+\frac{z^{2}}{k_{z}}}\right)}\;.$$

It is often more appropriate to represent the temperature distribution in the $\bar{x}$ - $\bar{y}$ - $\bar{z}$ coordinate system, which shares the same origin as the x - y - z system, and $\bar{z}$ remains coincident with z , while $\bar{x}$ is aligned along the velocity vector. In this scenario, a two-dimensional rotational coordinate transformation is applied to equation (35):

(36) $$T=T_{\infty }+\frac{Q}{4\pi \sqrt{k_{x}k_{y}k_{z}}\;\sqrt{\frac{{\left(\bar{x}\cos\;\theta -\bar{y}\;\sin\;\theta \right)}^{2}}{k_{x}}+\frac{{\left(\bar{x}\;\sin\;\theta +\bar{y}\cos\;\theta \right)}^{2}}{k_{y}}+\frac{{\bar{z}}^{2}}{k_{z}}}}\times e^{-\frac{1}{2}\rho c_{p}v\left(\frac{\left(\bar{x}\cos\;\theta -\bar{y}\;\sin\;\theta \right)\cos\;\theta }{k_{x}}+\frac{\left(\bar{x}\;\sin\;\theta +\bar{y}\cos\;\theta \right)\sin\;\theta }{k_{y}}+\sqrt{\frac{\cos^{2}\;\theta }{k_{x}}+\frac{\sin^{2}\;\theta }{k_{y}}}\;\sqrt{\frac{{\left(\bar{x}\cos\;\theta -\bar{y}\;\sin\;\theta \right)}^{2}}{k_{x}}+\frac{{\left(\bar{x}\;\sin\;\theta +\bar{y}\cos\;\theta \right)}^{2}}{k_{y}}+\frac{{\bar{z}}^{2}}{k_{z}}}\right)}\;.$$

The presented solution provides insight into the temperature distribution within an infinite solid, in which a moving point source with power Q travels, and where temperature is solely a function of spatial coordinates. This steady-state condition holds since the reference frame is attached to the heat source moving with constant velocity. The temperature at any given point remains constant in this moving frame, where the point is stationary with respect to the frame but moves relative to the material. However, when considering the thermal history of the material – where time-dependent properties (e.g., cooling-rate-dependent crystallization of thermoplastic polymers^22^) become relevant – the instantaneous rate of temperature change on the material can still be expressed within the moving reference frame, as detailed in Appendix B:

(37) $$\dot{T}=\frac{T-T_{\infty }}{\frac{{\left(\bar{x}\cos\;\theta -\bar{y}\;\sin\;\theta \right)}^{2}}{k_{x}}+\frac{{\left(\bar{x}\;\sin\;\theta +\bar{y}\cos\;\theta \right)}^{2}}{k_{y}}+\frac{{\bar{z}}^{2}}{k_{z}}}\times \left[v\left(\frac{\left(\bar{x}\cos\;\theta -\bar{y}\;\sin\;\theta \right)\cos\;\theta }{k_{x}}+\frac{\left(\bar{x}\;\sin\;\theta +\bar{y}\cos\;\theta \right)\sin\;\theta }{k_{y}}\right)+\frac{1}{2}\rho c_{p}v^{2}\times \left(\frac{\left(\bar{x}\cos\;\theta -\bar{y}\;\sin\;\theta \right)\cos\;\theta }{k_{x}}+\frac{\left(\bar{x}\;\sin\;\theta +\bar{y}\cos\;\theta \right)\sin\;\theta }{k_{y}}\right)\times \sqrt{\frac{\cos^{2}\;\theta }{k_{x}}+\frac{\sin^{2}\;\theta }{k_{y}}}\times \sqrt{\frac{{\left(\bar{x}\cos\;\theta -\bar{y}\;\sin\;\theta \right)}^{2}}{k_{x}}+\frac{{\left(\bar{x}\;\sin\;\theta +\bar{y}\cos\;\theta \right)}^{2}}{k_{y}}+\frac{{\bar{z}}^{2}}{k_{z}}}+\frac{1}{2}\rho c_{p}v^{2}\times \left(\frac{\cos^{2}\;\theta }{k_{x}}+\frac{\sin^{2}\;\theta }{k_{y}}\right)\times \left(\frac{{\left(\bar{x}\cos\;\theta -\bar{y}\;\sin\;\theta \right)}^{2}}{k_{x}}+\frac{{\left(\bar{x}\;\sin\;\theta +\bar{y}\cos\;\theta \right)}^{2}}{k_{y}}+\frac{{\bar{z}}^{2}}{k_{z}}\right)\;\right]\;.$$

The surface centerline ( $\bar{y}=0,\bar{z}=0$ ) is often of particular interest in practice for evaluating representative temperature distribution and representative heating and cooling rates. This condition simplifies equations (36) and (37) to the following forms, respectively:

(38) $$T=T_{\infty }+\frac{Q}{4\pi \sqrt{k_{x}k_{y}k_{z}}\;\sqrt{\;\frac{\cos^{2}\;\theta }{k_{x}}+\frac{\sin^{2}\;\theta }{k_{y}}}\;\bar{x}}\;e^{-\rho \;c_{p}v\;\left(\;\frac{\cos^{2}\;\theta }{k_{x}}+\frac{\sin^{2}\;\theta }{k_{y}}\right)\;\bar{x}},$$

(39) $$\dot{T}=\left(T-T_{\infty }\right)\times \left[\frac{v}{\bar{x}}+\rho c_{p}v^{2}\left(\frac{\cos^{2}\;\theta }{k_{x}}+\frac{\sin^{2}\;\theta }{k_{y}}\right)\right]\;.$$

Returning to equation (36), while the developed closed-form solution represents the temperature distribution resulting from a point heat source moving within an infinite orthotropic solid, due to the symmetry of heat propagation with respect to the $\bar{x}$ *-* $\bar{y}$ plane ( $\bar{z}=0$ ), the same formulation can be applied to model the temperature distribution induced by a point heat source injecting power $Q^{\prime }=Q/2$ , moving along the adiabatic surface of a semi-infinite orthotropic solid. Therefore, the term Q can be simply replaced by $2Q^{\prime }$ to adapt the solution for a moving heat source with power $Q^{\prime }$ on the surface of a semi-infinite solid. The verification of this analytical model using finite element analysis will be subsequently discussed.

### Orthotropic Solids with finite Thickness

The next step involves evaluating the effect of a moving heat source on solids with finite thickness. For constant and known velocity, material properties, and orientation, as shown in equation (36), the temperature distribution can be represented by the elementary function f :

(40) $$T-T_{\infty }=f\left(Q,\bar{x},\bar{y},\bar{z}\right)\;.$$

**Figure 4.**
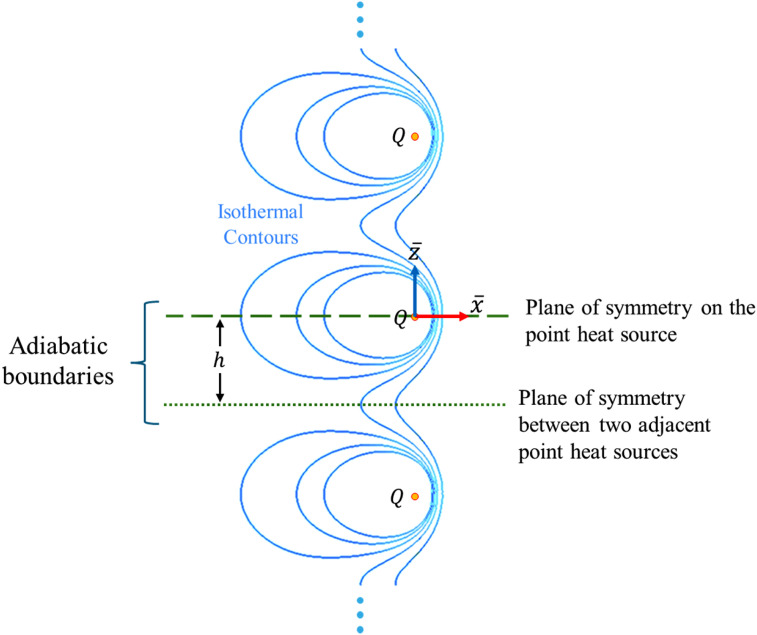
Vertically aligned multiple identical point heat sources and corresponding planes of symmetry.

As a linear system, the temperature distribution in the solid domain induced by multiple heat sources can be obtained by summation of the effects of all individual sources. Consider an infinite number of point heat sources, spaced 2h apart along the $\bar{z}$ axis, each with identical power and velocity vectors, moving through an infinite solid as depicted in Figure 4. The resulting temperature distribution is given by:

(41) $$T-T_{\infty }=\overset{K}{\underset{\kappa =-K}{\sum }}f\left(Q,\bar{x},\bar{y},\bar{z}+2\kappa h\right),K\to \infty .$$

In this configuration, two sets of symmetry planes emerge, functioning as adiabatic boundaries due to the zero heat flux across symmetry planes. The first set consists of horizontal planes passing through the point heat source, one of which is depicted by a dashed line in Figure 4. The second set includes horizontal planes midway between two adjacent heat sources, one of which is shown as a dotted line in Figure 4. The domain $-h\leq \bar{z}\leq 0$ can effectively represent a solid of finite thickness with adiabatic top and bottom surfaces, on which a moving point heat source with power Q/2 injects heat into the system at the location ( $\bar{x}=0,\bar{y}=0,\bar{z}=0$ ).

### Two-dimensional Solution

Although the primary focus of this study is not on two-dimensional planar heat transfer, such analysis becomes relevant in certain applications – such as thin plates – where through-thickness thermal gradients are insignificant and in-plane heat conduction dominates. An exact two-dimensional solution cannot be obtained as a limiting case of equation (36). Instead, the governing equations should be derived independently, as presented in Appendix C. The final expression for the temperature field is:

(42) $$T=T_{\infty }+\frac{\bar{Q}}{2\pi \sqrt{k_{x}k_{y}}\;}\left[e^{-\frac{1}{2}\rho c_{p}v\left(\frac{\left(\bar{x}\cos\;\theta -\bar{y}\;\sin\;\theta \right)\cos\;\theta }{k_{x}}+\frac{\left(\bar{x}\;\sin\;\theta +\bar{y}\cos\;\theta \right)\sin\;\theta }{k_{y}}\right)}\right]\times K_{0}\left(\frac{1}{2}\rho c_{p}v\;\sqrt{\frac{\cos^{2}\;\theta }{k_{x}}+\frac{\sin^{2}\;\theta }{k_{y}}}\;\sqrt{\frac{{\left(\bar{x}\cos\;\theta -\bar{y}\;\sin\;\theta \right)}^{2}}{k_{x}}+\frac{{\left(\bar{x}\;\sin\;\theta +\bar{y}\cos\;\theta \right)}^{2}}{k_{y}}}\right).$$

Here, $\bar{Q}$ denotes the heat source power per unit depth [W/m], and $K_{0}$ represents the modified Bessel function of the second kind of order zero.

## Verification

Finite element analysis was performed using COMSOL Multiphysics, employing quadratic Lagrange elements for temperature discretization. In the first step, to perform verification of the analytical model for a semi-infinite solid, the model geometry consisted of a rectangular solid block with dimensions 0.2 m × 0.05 m × 0.01 m. These dimensions were chosen large enough to ensure that the region of interest around the point heat source remained unaffected by boundary effects, thereby satisfying the assumption of a semi-infinite solid. Boundary conditions were specified as follows: constant room temperature was applied to the two lateral faces, the bottom face, and the front (upstream) face, while thermal insulation was imposed on the top face and the back (downstream) face. A point heat source was positioned at the center of the top face (origin) to inject a constant heat power into the system. The relative motion of the heat source was incorporated by defining a translational motion for the solid domain with velocity field of −v in the $\bar{x}$ direction to enable steady-state analysis. In this configuration, the point heat source was treated as stationary within the computational domain, while capturing a relative velocity of v in the $\bar{x}$ direction with respect to the solid.

**Figure 5.**
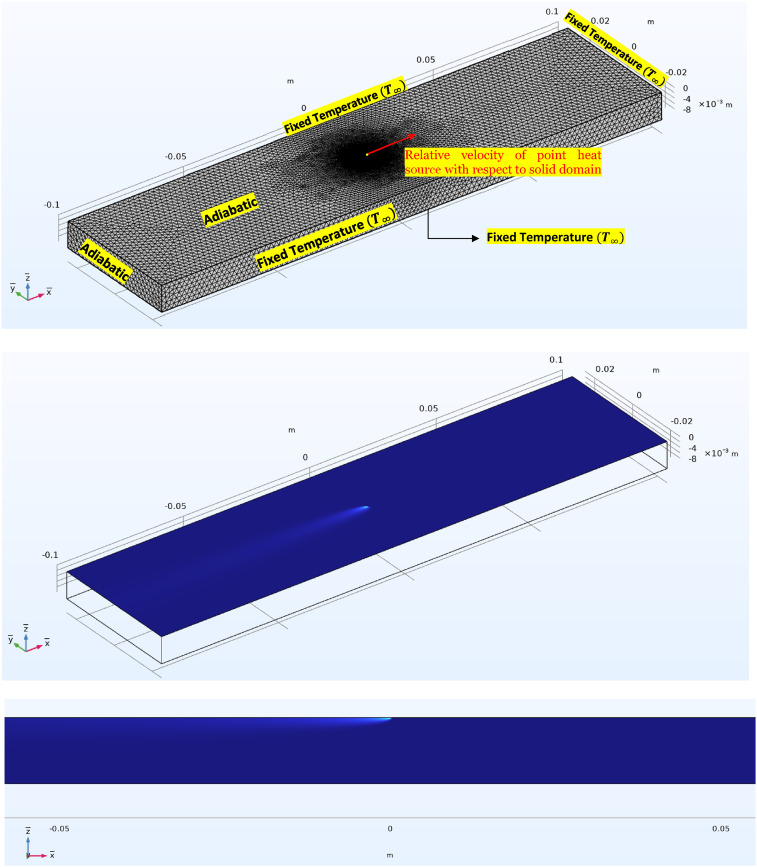
Finite element model in COMSOL Multiphysics.

The mesh was constructed using tetrahedral elements. Mesh parameters included a maximum element size of 2 × 10^−3^ m, a minimum element size of 1 × 10^−6^ m, and a maximum growth rate of 1.05. This meshing strategy resulted in a total of 1,373,879 elements, with the highest mesh resolution concentrated in the vicinity of the point heat source to capture critical thermal gradients, while coarser resolution was employed in regions distant from the area of interest to optimize computational efficiency (see Figure 5 (top)). The input parameters of the simulation are as follows, where the selected material as our example is Carbon-Fiber/Poly-Ether-Ether-Ketone (CF/PEEK) (Table 1):

**Table 1.** Values of CF/PEEK thermal properties^23^ and parameters used for FEM verification.

Parameters	Values
$k_{x}$	5.9 W/(m*K)
$k_{y}$	0.7 W/(m*K)
$k_{z}$	0.7 W/(m*K)
ρ	1575 kg/(m^3)
$c_{p}$	1300 J/(kg*K)
v	1 cm/s
$Q^{\prime }$	2 W
$T_{\infty }$	20**°**C
θ	0**°**, 45**°**, 90**°**

Figure 5 (middle) and (bottom) present color maps of the temperature distribution calculated using the FEM in the $\bar{x}$ - $\bar{y}$ and $\bar{x}$ - $\bar{z}$ planes, respectively. These results confirm that the block’s width and thickness were sufficiently large to replicate heat propagation in a semi-infinite solid within the region of interest around the point heat source.

**Figure 6.**
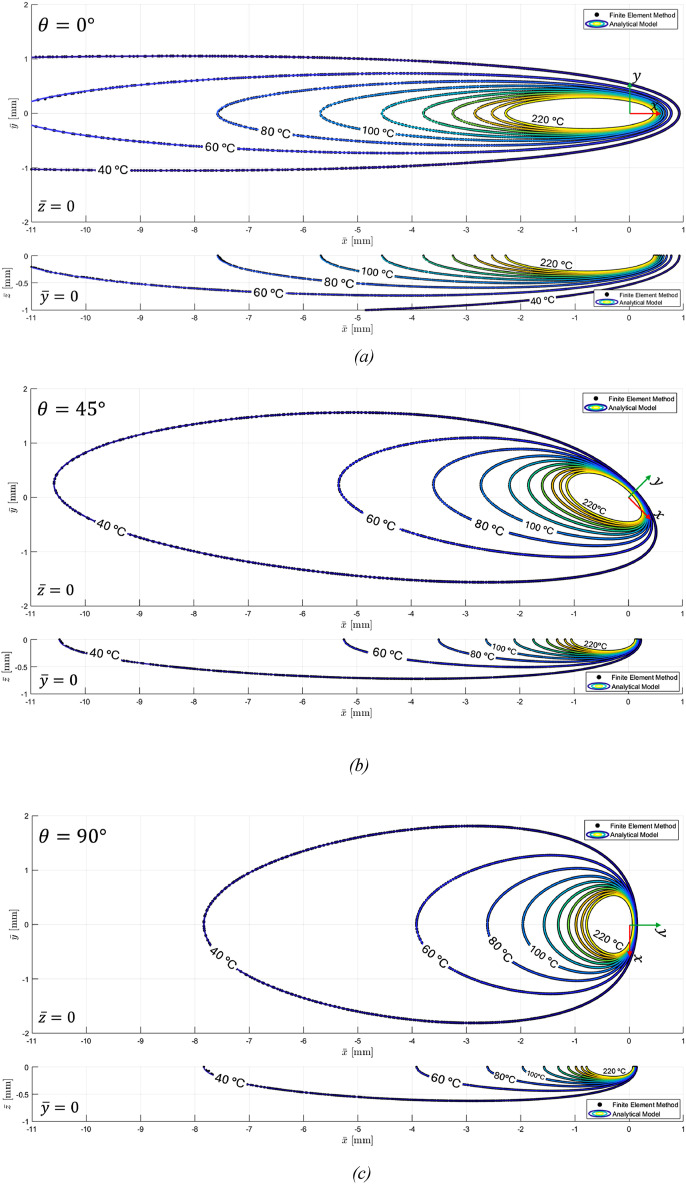
(a). Temperature distributions from analytical solution and FEM for a semi-infinite solid with heat source velocity oriented at 0° to the material x-axis: in-plane (top) and through-thickness (bottom). (b). Temperature distributions from analytical solution and FEM for a semi-infinite solid with heat source velocity oriented at 45° to the material x-axis: in-plane (top) and through-thickness (bottom). (c). Temperature distributions from analytical solution and FEM for a semi-infinite solid with heat source velocity oriented at 90° to the material x-axis: in-plane (top) and through-thickness (bottom).

The results of the analytical model for a semi-infinite solid and the finite element analysis are compared in Figure 6, represented as isothermal contours for three different orientations of the velocity vector relative to the material’s principal x-axis. For each orientation, temperature distributions in the $\bar{x}$ - $\bar{y}$ and $\bar{x}$ - $\bar{z}$ planes are presented. The FEM results are shown as discrete data points, while the analytical solution is depicted using continuous colored lines. The excellent agreement between the two approaches confirms the validity of equation (36). The contours indicate that when the velocity vector aligns with one of the material’s principal axes (e.g., aligned with *x* ( θ=0° ) or with *y* ( θ=90° )), the temperature distribution exhibits symmetry with respect to the $\bar{x}$ - $\bar{z}$ plane. In contrast, when the heat source is moving off-axis (e.g., θ = 45°), the temperature distribution becomes asymmetric relative to the $\bar{x}$ - $\bar{z}$ plane.

**Figure 7.**
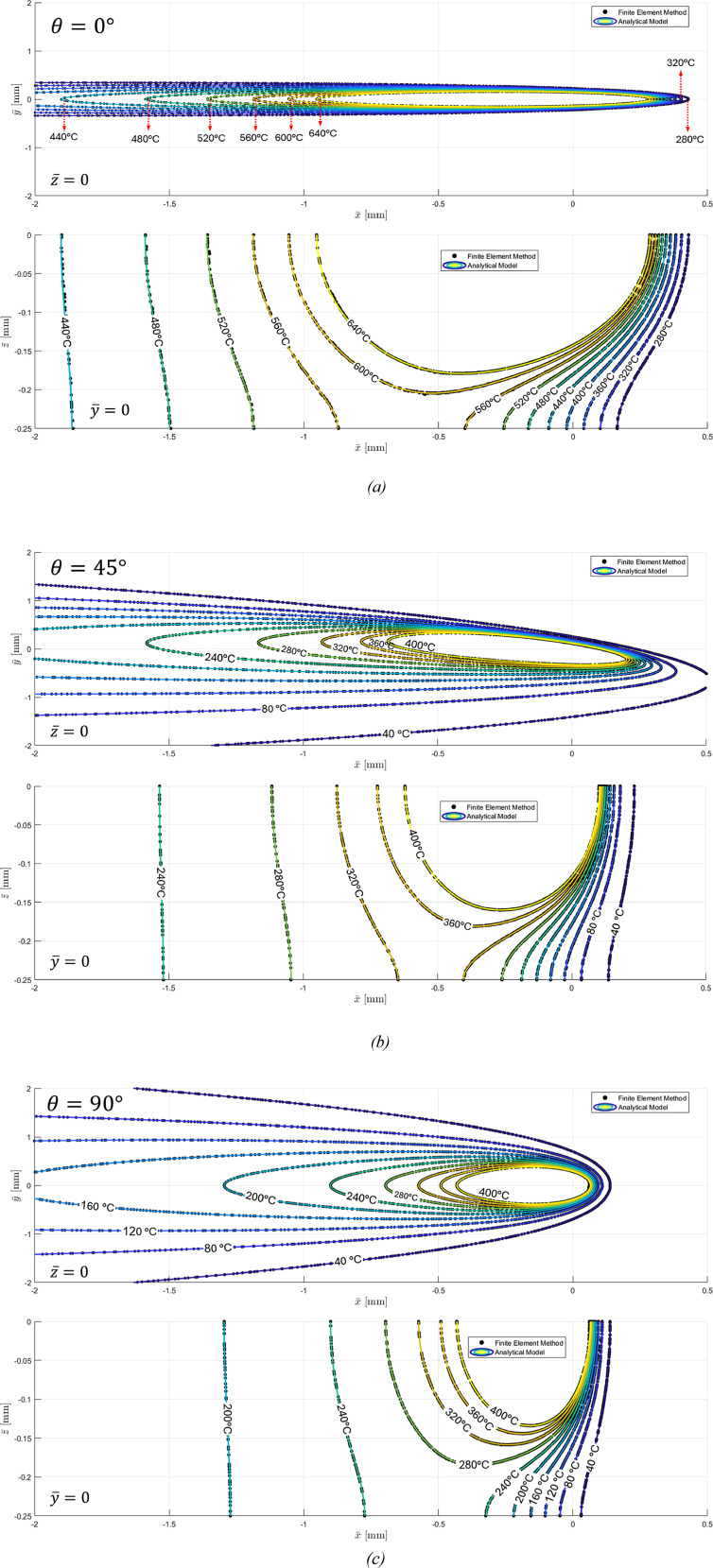
(a). Temperature distributions from analytical solution and FEM for a finite-thickness solid with heat source velocity oriented at 0° to the material x-axis: in-plane (top) and through-thickness (bottom). (b). Temperature distributions from analytical solution and FEM for a finite-thickness solid with heat source velocity oriented at 45° to the material x-axis: in-plane (top) and through-thickness (bottom). (c). Temperature distributions from analytical solution and FEM for a finite-thickness solid with heat source velocity oriented at 90° to the material x-axis: in-plane (top) and through-thickness (bottom).

In another stage of verification, the analytical model for a solid with finite thickness (equation (41)) was compared with finite element analysis (FEA). For this case, the geometry of the FEA model was modified by significantly reducing the thickness to 0.25 × 10^−3^ m to account for the influence of the bottom face on the temperature distribution. The boundary condition at the bottom face was then set to adiabatic, while all other parameters, including those listed in Table 1, remained unchanged. The comparison of the analytical and FEA results for the in-plane and through-thickness temperature distributions are illustrated in Figure 7 for three different orientations of the material’s x-axis relative to the heat source velocity. Once again, the excellent agreement between the analytical and FEA results confirms the validity of the analytical framework. In the analytical calculations for this case, the parameter *K* in equation (41) was set to only 5 (superposition of 11 heat sources), which proved sufficient to achieve high accuracy in the region of interest around the heat source. Due to the exponential decay of the heat source’s effect with distance, selecting a large value for *K* is unnecessary to maintain acceptable accuracy.

## Distributed heat source

The theoretical framework for thermal analysis using a moving point heat source is only valid in practical applications when the heating system delivers highly localized power to the surface. This imposes a limitation on the direct implementation of the existing analytical solution for practical systems. To overcome this constraint, the surface area of the distributed heat flux, which effectively governs the temperature distribution within the solid, is discretized into a series of point heat sources. This discretization aims to replicate the thermal influence of a continuous heat flux distribution. Leveraging the linearity of the thermal system, the temperature at any point within the solid domain can be computed as the superposition of the temperature increments (ΔT) induced by each individual point heat source.

**Figure 8.**
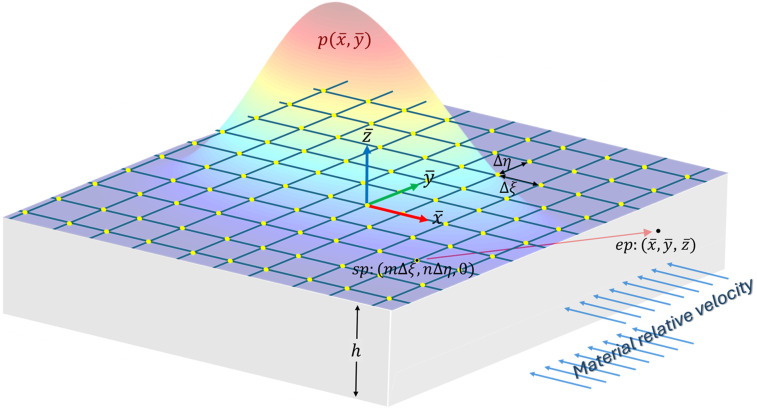
Representation of a continuous heat source as an array of discrete point sources.

Let $p\left(\bar{x},\bar{y}\right)$ represent the given continuous heat flux profile, with the origin of $\bar{x}$ - $\bar{y}$ - $\bar{z}$ coordinate system positioned at the peak of p . The effective surface area of the heat flux can be discretized into a grid of $\left(M_{f}+M_{b}+1\right)\times \left(N_{l}+N_{r}+1\right)$ point heat sources, spaced at intervals of Δξ in the $\bar{x}$ -direction and Δη in the $\bar{y}$ -direction, as illustrated in Figure 8.

The temperature at an arbitrary excited point (*ep*) located at $(\bar{x},\bar{y}$ , $\bar{z})$ within an orthotropic solid with an adiabatic bottom surface can be determined by summing the temperature increments induced by all source points (*sp*). This can be expressed as:

(43) $$T-T_{\infty }=\overset{M_{f}}{\underset{m=-M_{b}}{\sum }}\;\overset{N_{l}}{\underset{n=-N_{r}}{\sum }}\;\overset{K}{\underset{\kappa =-K}{\sum }}f\left(p_{\left(m∆\xi ,n∆\eta \right)}\Delta \xi \Delta \eta ,\bar{x}-m∆\xi ,\bar{y}-n∆\eta ,\bar{z}+2\kappa h\right)\;.$$

The bounds of the summation indices, m (ranging from $-M_{b}$ ​ to $M_{f}$ ​) and n (ranging from $-N_{r}$ ​ to $N_{l}$ ​), which define the computational domain, need to be determined with an understanding of the heat flux distribution profile. The spatial extent of the summation, represented as a rectangular area with dimensions $\left(M_{f}+M_{b}\right)\Delta \xi \times \left(N_{l}+N_{r}\right)\Delta \eta$ , should be sufficiently large to capture the region responsible for at least 99% of the total heat exchange with the surface.

An additional consideration in defining the computational domain is the choice of Δξ and Δη , which need to be sufficiently small to avoid significant truncation errors where steep gradients of the heat distribution exist. Another critical consideration in selecting the sizes of Δξ and Δη arises when it comes to the location of target excited point for temperature calculation. When the excited point is located closer to the surface, the discrete source points must be positioned closer together (i.e., smaller Δξ and Δη ) to more effectively replicate the effects of a continuous heating profile. This requirement will be clarified in the following example of calculations.

Even though the heat flux profile $p\left(\bar{x},\bar{y}\right)$ could be any arbitrary function, including non-elementary functions (e.g., a piecewise function), or discrete sets of data points, in this example we adopt a 2D Gaussian function with elliptical decay as the heat source which can approximate a laser beam or a light source:

(44) $$p=p_{\max}\;e^{-\left(\frac{{\bar{x}}^{2}}{a^{2}}+\frac{{\bar{y}}^{2}}{b^{2}}\;\right)}\;.$$

The constants $p_{\max}$ , a , and b selected in the upcoming analysis are listed in Table 2. The thickness and material orientation are also presented in Table 2, while the selected material properties and the velocity of the heat source are the same as those presented in Table 1.

**Table 2.** Parameters and corresponding values used for FEM verification.

Parameters	Values
$p_{\max}$	6E5 W/(m^ 2)
a	5E-3 m
b	2E-3 m
h	2.5E-3 m
θ	90°

**Figure 9.**
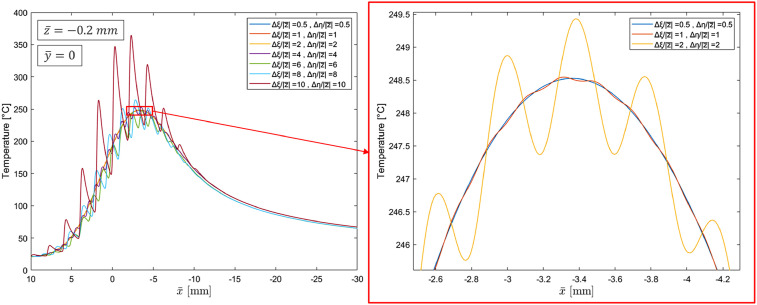
Predicted temperature distribution along the axis of motion for different point source spacing ratios.

Calculations were performed at 3000 points within the solid, along a line segment spanning from (10 mm, 0 mm, −0.2 mm) to (−30 mm, 0 mm, −0.2 mm), for various heat source spacings ( Δξ = Δη ) of 0.1 mm, 0.2 mm, 0.4 mm, 0.8 mm, 1.2 mm, 1.6 mm, and 2.0 mm. The resulting temperature distributions, as shown in Figure 9, indicate that when the source spacing becomes too large relative to the depth of the target point ( $\bar{z})$ , significant fluctuations emerge. These fluctuations, which stem from the nature of discrete point sources, deviate from the continuous nature of the actual heat flux profile. An analysis across different source spacings, depths, and heat flux intensities (Figure 9 presents results for a single depth only) suggests that for achieving high accuracy, defined as an amplitude of fluctuation waves less than 0.1% of local magnitude of ( $T-T_{\infty })$ , the following condition must be satisfied for the spacing size:

(45) $$\max\left(\frac{\Delta \xi }{\left|\bar{z}\right|},\frac{\Delta \eta }{\left|\bar{z}\right|}\right)\leq 1\;.$$

**Figure 10.**
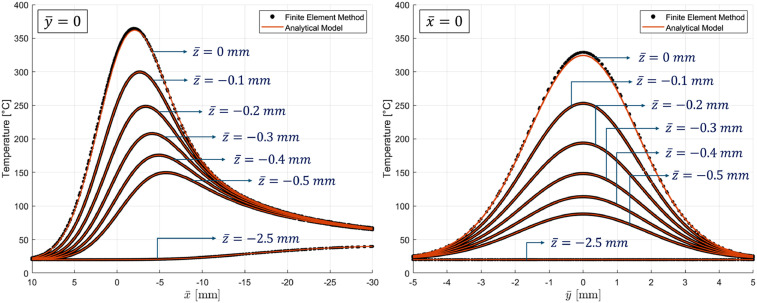
Temperature distribution along (left plot) and perpendicular (right plot) to the direction of source motion: Validation of the model for a distributed heat source against FEM results at different depths.

In Figure 10, the temperature distributions predicted by the proposed model are shown at various depths within the $\bar{x}-\bar{z}$ plane and $\bar{y}-\bar{z}$ plane and are compared with finite element simulation results with a continuous heat flux profile on the surface using equation (44) and parameters in Table 2. To maintain computational efficiency, the spatial discretization ratios are set as $\frac{\Delta \xi }{\left|\bar{z}\right|}=\frac{\Delta \eta }{\left|\bar{z}\right|}=1$ . It is worthy of attention that direct computation of the temperature distribution at the surface ( $\bar{z}=0$ ) is not feasible since Δξ and Δη must approach zero to achieve sufficient resolution. To overcome this, the surface temperature can be determined by extrapolating temperature values from deeper layers. For example, linear extrapolation using data at $\bar{z}=-d$ and $\bar{z}=-d/2$ can approximate the temperature at $\bar{z}=0$ . The accuracy of the extrapolation depends on the choice of d , which needs to be selected small enough to ensure capturing the variations effectively for high accuracy at regions with steep gradient. A practical guideline for the size of d is to ensure that:

(46) $$\frac{\left|T\left(\bar{x},\bar{y},-\frac{d}{2}\right)-T\left(\bar{x},\bar{y},-d\right)\right|}{T\left(\bar{x},\bar{y},-\frac{3d}{4}\right)-T_{\infty }}<10\%\;.$$

For example, the predicted surface temperature results shown in Figure 10 are obtained by extrapolating the temperature curves at $\bar{z}=-0.1$ mm and $\bar{z}=-0.05$ mm to $\bar{z}=0$ . In the presented results, the error in the predicted surface temperature using extrapolation is below 2%, whereas the directly computed temperatures for deeper layers exhibit errors below 0.1%.

The steady-state simulation computation time for the COMSOL finite element solver on a typical personal computer was measured at 38 seconds, while the computation time for each point using the analytical model was on the order of centi-seconds (e.g., 0.029 seconds for $\frac{\Delta \xi }{\left|\bar{z}\right|}$ = 1) on the same computer. This can highlight the utility of the analytical approach for rapid temperature estimations when the purpose is to determine temperature at a specific point or along lines consisting of a moderate number of points. The finite element solver in COMSOL solves a large system of algebraic equations derived from the discretized heat transfer equations using advanced iterative methods, such as the GCRO-DR (Generalized Conjugate Residual with Deflated Restarts) method, being specifically optimized for computational efficiency in solving large linear systems, making it well-suited for the current linear simulation. However, while the finite element method requires the definition of boundary conditions over a domain that extends far beyond the region of interest to ensure accurate results, the analytical approach evades this dependency. The analytical solution directly computes the temperature at desired locations without being influenced by the solution for adjacent points or the requirement of defining far-field conditions. The independence from boundary condition definitions in remote regions and the ability to compute temperature directly at a target point, simplifies the analytical approach and enhances its practicality. In the meantime, the small computation time may show potential for real-time thermal predictions when it comes to smart process control systems in continuous composite manufacturing/joining processes, e.g., AFP or induction welding, which enables dynamic adjustments to process parameters to maintain operations within the optimal window.

**Figure 11.**
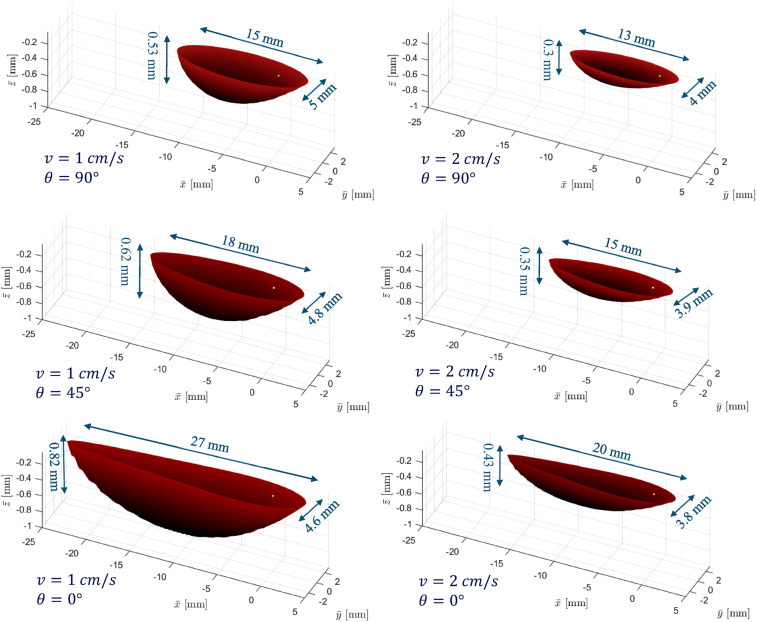
Iso-surface of T = 143 ℃ for different heat source velocities and material orientations depicted with relative [ $\bar{x}$ :1, $\bar{y}$ :1, $\bar{z}$ :10] scale of magnification (yellow point marking the origin of the reference frame, i.e., the center of the heat source profile).

A parametric analysis comparing different fiber orientations and heat source velocities under the same distributed heating condition (Table 2) and material properties (Table 1) is presented in Figure 11. The results are visualized using the iso-surface corresponding to the critical glass transition temperature (Tg = 143 ℃ ) of CF/PEEK, with the maximum width, length, and depth of the heat-soaked region annotated for each case.

The results indicate that at a velocity of 1 cm/s, reducing the fiber angle from 90° to 0° leads to a moderate reduction (8%) in the width of the region exceeding Tg ​, while the length of this region increases substantially (80%). A similar trend is observed at 2 cm/s, where the same variation in fiber orientation results in a 5% reduction in width and a 54% increase in length. Regarding the maximum penetration depth of the heated region, decreasing the fiber angle from 90° to 0° results in a 55% increase at 1 cm/s and a 43% increase at 2 cm/s. These findings suggest that as the axis of maximum thermal conductivity (i.e., typically fiber direction for CFRPs) becomes more parallel to the direction of heat source motion, the thermally affected zone becomes not only longer but also deeper. Furthermore, at lower velocities, the dimensions of the heated zone exhibit greater sensitivity to fiber orientation than at higher velocities.

From a different standpoint, increasing the heat source velocity from 1 cm/s to 2 cm/s reduces the size of the region, as the material is exposed to the heat source for a shorter duration. The reduction in length is quantified as 13%, 17%, and 26% for fiber angles of 90°, 45°, and 0°, respectively, while the corresponding reductions in width are 20%, 19%, and 17%. The most pronounced effect is observed in depth reduction, which decreases by 43%, 44%, and 48% for fiber angles of 90°, 45°, and 0°, respectively. Overall, while this analysis highlights the influence of fiber orientation and velocity on the dimensions of the heat-soaked region, the sensitivity of these effects is directly proportional to the material’s degree of orthotropy (e.g., $k_{x}/k_{y}$ ) in case of a general material.

## Conclusion

This paper presents a closed-form solution for the thermal analysis of a moving point heat source over a semi-infinite orthotropic solid with arbitrary orientation (e.g., off-axis motion). The resulting temperature distribution is expressed in equation (36), while the local rate of temperature change in the material is formulated in equation (37). While equation (36) was derived for a point heat source of power Q moving within an infinite solid, it remains directly applicable to the case of a moving heat source of power $Q^{\prime }=Q/2$ on the surface of a semi-infinite solid, as a result of symmetry considerations. The solution is then extended to account for finite-thickness solids with adiabatic boundaries, broadening its applicability to practical scenarios. The accuracy of the proposed solution has been verified through very close agreement with results from finite element analysis. Furthermore, a discretization methodology is introduced to approximate continuous heat sources with arbitrary shapes and distributions by superimposing multiple point heat sources, which has also been validated against the finite element method to prove its excellent reliability. The proposed theoretical framework offers a computationally efficient approach for rapid thermal predictions, making it applicable to simulating thermal distributions in the processing of orthotropic composite materials. Finally, although the primary focus of this work was the development of a three-dimensional analytical framework, a two-dimensional solution was also derived and succinctly presented in equation (42), offering potential applicability to thin-plate scenarios.

## Figures and Tables

**Figure C1.**
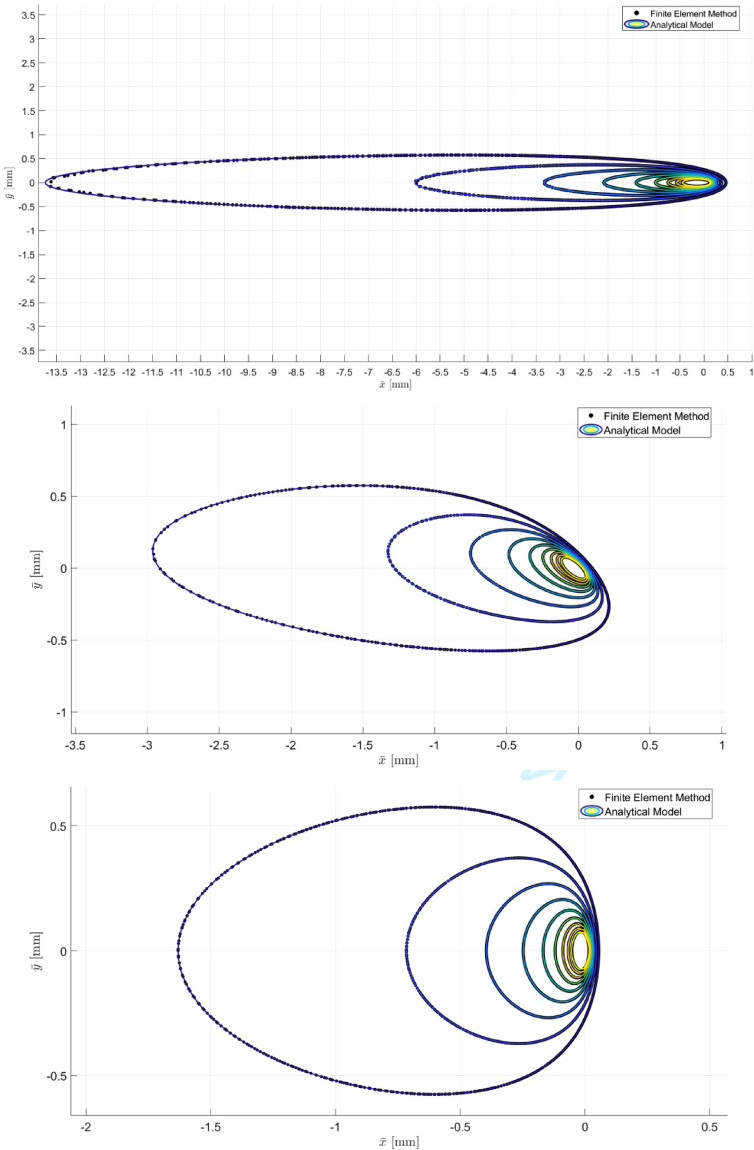
Comparison between the 2D analytical solution and 2D FEM results for various material orientations relative to the heat source velocity (θ = 0°, 45°, and 90° from top to bottom). Isothermal contours are shown from T = 60°C to T = 220°C with a temperature interval of 20°C.

## Appendix

### Appendix A (derivations for equation (20))

Equation (18) can be shown as:

(A1)
$$\frac{\partial^{2}T}{\partial X^{2}}+\frac{\partial^{2}T}{\partial Y^{2}}+\frac{\partial^{2}T}{\partial Z^{2}}+\rho c_{p}V_{x}\frac{\partial T}{\partial X}+\rho c_{p}V_{y}\frac{\partial T}{\partial Y}=0\;.$$

Also, equation (19) can be shown as:

(A2)
$$T=T_{\infty }+e^{-\frac{1}{2}\rho c_{p}V_{x}X}e^{-\frac{1}{2}\rho c_{p}V_{y}Y}\varphi \left(X,Y,Z\right)\;.$$

Based on the product rule for partial derivatives:

(A3)
$$\frac{\partial T}{\partial X}=-\frac{1}{2}\rho c_{p}V_{x}\;e^{-\frac{1}{2}\rho c_{p}V_{x}X}e^{-\frac{1}{2}\rho c_{p}V_{y}Y}\varphi +e^{-\frac{1}{2}\rho c_{p}V_{x}X}e^{-\frac{1}{2}\rho c_{p}V_{y}Y}\frac{\partial \varphi }{\partial X}\;.$$

Now, the exponential terms are factored out from equation (A3):

(A4)
$$\frac{\partial T}{\partial X}=\left(-\frac{1}{2}\rho c_{p}V_{x}\;\varphi +\frac{\partial \varphi }{\partial X}\right)e^{-\frac{1}{2}\rho c_{p}V_{x}X}e^{-\frac{1}{2}\rho c_{p}V_{y}Y}\;.$$

With taking another derivative with respect to *X*, the following is obtained:

(A5)
$$\frac{\partial^{2}T}{\partial X^{2}}=\left(-\frac{1}{2}\rho c_{p}V_{x}\;\frac{\partial \varphi }{\partial X}+\frac{\partial^{2}\varphi }{\partial X^{2}}\right)e^{-\frac{1}{2}\rho c_{p}V_{x}X}e^{-\frac{1}{2}\rho c_{p}V_{y}Y}+\left(-\frac{1}{2}\rho c_{p}V_{x}\;\varphi +\frac{\partial \varphi }{\partial X}\right)\left(-\frac{1}{2}\rho c_{p}V_{x}\;\right)e^{-\frac{1}{2}\rho c_{p}V_{x}X}e^{-\frac{1}{2}\rho c_{p}V_{y}Y}\;.$$

By factoring out exponential terms, it appears as:

(A6)
$$\frac{\partial^{2}T}{\partial X^{2}}=\left({\left(-\frac{1}{2}\rho c_{p}V_{x}\right)}^{2}\varphi -\rho c_{p}V_{x}\;\frac{\partial \varphi }{\partial X}+\frac{\partial^{2}\varphi }{\partial X^{2}}\right)e^{-\frac{1}{2}\rho c_{p}V_{x}X}e^{-\frac{1}{2}\rho c_{p}V_{y}Y}\;.$$

Let’s multiply both sides of equation (A4) by $\rho c_{p}V_{x}$ :

(A7)
$$\rho c_{p}V_{x}\frac{\partial T}{\partial X}=\rho c_{p}V_{x}\left(-\frac{1}{2}\rho c_{p}V_{x}\;\varphi +\frac{\partial \varphi }{\partial X}\right)e^{-\frac{1}{2}\rho c_{p}V_{x}X}e^{-\frac{1}{2}\rho c_{p}V_{y}Y}\;.$$

By summing equation (A6) and equation (A7), we arrive at:

(A8)
$$\frac{\partial^{2}T}{\partial X^{2}}+\rho c_{p}V_{x}\frac{\partial T}{\partial X}=\left[\frac{\partial^{2}\varphi }{\partial X^{2}}-{\left(\frac{1}{2}\rho c_{p}V_{x}\right)}^{2}\varphi \right]e^{-\frac{1}{2}\rho c_{p}V_{x}X}e^{-\frac{1}{2}\rho c_{p}V_{y}Y}\;.$$

Similarly, the same procedure is performed to derive partial derivatives with respect to *Y*:

(A9)
$$\frac{\partial^{2}T}{\partial Y^{2}}+\rho c_{p}V_{y}\frac{\partial T}{\partial Y}=\left[\frac{\partial^{2}\varphi }{\partial Y^{2}}-{\left(\frac{1}{2}\rho c_{p}V_{y}\right)}^{2}\varphi \right]e^{-\frac{1}{2}\rho c_{p}V_{x}X}e^{-\frac{1}{2}\rho c_{p}V_{y}Y}\;.$$

Moreover, the second derivative of *T* with respect to *Z*, which can be simply derived from equation (A2), will be:

(A10)
$$\frac{\partial^{2}T}{\partial Z^{2}}=\left[\frac{\partial^{2}\varphi }{\partial Z^{2}}\right]e^{-\frac{1}{2}\rho c_{p}V_{x}X}e^{-\frac{1}{2}\rho c_{p}V_{y}Y}\;.$$

By summing up equations (A8), (A9), and (A10), the following equation is obtained:

(A11)
$$\frac{\partial^{2}T}{\partial X^{2}}+\frac{\partial^{2}T}{\partial Y^{2}}+\frac{\partial^{2}T}{\partial Z^{2}}+\rho c_{p}V_{x}\frac{\partial T}{\partial X}+\rho c_{p}V_{y}\frac{\partial T}{\partial Y}=\left[\frac{\partial^{2}\varphi }{\partial X^{2}}-{\left(\frac{1}{2}\rho c_{p}V_{x}\right)}^{2}\varphi +\frac{\partial^{2}\varphi }{\partial Y^{2}}-{\left(\frac{1}{2}\rho c_{p}V_{y}\right)}^{2}\varphi +\frac{\partial^{2}\varphi }{\partial Z^{2}}\right]e^{-\frac{1}{2}\rho c_{p}V_{x}X}e^{-\frac{1}{2}\rho c_{p}V_{y}Y}.$$

It can be seen that the left side of equation (A11) is the same as the left side of equation (A1). Therefore:

(A12)
$$\left[\frac{\partial^{2}\varphi }{\partial X^{2}}-{\left(\frac{1}{2}\rho c_{p}V_{x}\right)}^{2}\varphi +\frac{\partial^{2}\varphi }{\partial Y^{2}}-{\left(\frac{1}{2}\rho c_{p}V_{y}\right)}^{2}\varphi +\frac{\partial^{2}\varphi }{\partial Z^{2}}\right]e^{-\frac{1}{2}\rho c_{p}V_{x}X}e^{-\frac{1}{2}\rho c_{p}V_{y}Y}=0\;.$$

Only the expression in the bracket can equal zero. Hence:

(A13)
$$\frac{\partial^{2}\varphi }{\partial X^{2}}+\frac{\partial^{2}\varphi }{\partial Y^{2}}+\frac{\partial^{2}\varphi }{\partial Z^{2}}-{\left(\frac{1}{2}\rho c_{p}\sqrt{{V_{x}}^{2}+{V_{y}}^{2}}\right)}^{2}\varphi =0\;.$$

### Appendix B (derivations for equation (37))

From equations (26) and (33), it is known that:

(B1)
$$\varphi =\frac{Q}{4\pi \sqrt{k_{x}k_{y}k_{z}}\;s}e^{-\frac{1}{2}\rho c_{p}\sqrt{{V_{x}}^{2}+{V_{y}}^{2}}\;s}\;.$$

First, let’s express the first partial derivative of φ with respect to s :

(B2)
$$\frac{\partial \varphi }{\partial s}=\frac{Q}{4\pi \sqrt{k_{x}k_{y}k_{z}}\;}\left(-\frac{1}{s^{2}}-\frac{1}{2s}\rho c_{p}\sqrt{{V_{x}}^{2}+{V_{y}}^{2}}\;\right)e^{-\frac{1}{2}\rho c_{p}\sqrt{{V_{x}}^{2}+{V_{y}}^{2}}\;s}\;.$$

Also, based on the definition of s in equation (21):

(B3)
$$\frac{\partial s}{\partial X}=\frac{X}{s}\;.$$

Using (B2) and (B3), the derivative of φ with respect to X will be:

(B4)
$$\frac{\partial \varphi }{\partial X}=\frac{Q}{4\pi \sqrt{k_{x}k_{y}k_{z}}\;s\;}\left(-\frac{X}{s^{2}}-\frac{X}{2s}\rho c_{p}\sqrt{{V_{x}}^{2}+{V_{y}}^{2}}\;\right)e^{-\frac{1}{2}\rho c_{p}\sqrt{{V_{x}}^{2}+{V_{y}}^{2}}\;s}\;.$$

By plugging (B1) and (B4) into (A4), we arrive at:

(B5)
$$\frac{\partial T}{\partial X}=\left(-\frac{X}{s^{2}}-\frac{X}{2s}\rho c_{p}\sqrt{{V_{x}}^{2}+{V_{y}}^{2}}-\frac{1}{2}\rho c_{p}V_{x}\right)\frac{Q}{4\pi \sqrt{k_{x}k_{y}k_{z}}\;s}e^{-\frac{1}{2}\rho c_{p}\left(V_{x}X+V_{y}Y+\sqrt{{V_{x}}^{2}+{V_{y}}^{2}}\;s\right)}\;.$$

The same procedure is applied to derive $\frac{\partial T}{\partial Y}$ , which will ultimately take a form similar to (B5), with the terms X and the second $V_{x}$ replaced by Y and $V_{y}$ , respectively. Meanwhile, to derive the rate of temperature change at an arbitrary stationary point within the solid, the following relation holds:

(B6)
$$\frac{\partial T}{\partial t}=\frac{\partial T}{\partial x}\frac{dx}{dt}+\frac{\partial T}{\partial y}\frac{dy}{dt}+\frac{\partial T}{\partial z}\frac{dz}{dt}=-v_{x}\frac{\partial T}{\partial x}-v_{y}\frac{\partial T}{\partial y}=-V_{x}\frac{\partial T}{\partial X}-V_{y}\frac{\partial T}{\partial Y}\;.$$

By substituting (B5) and its counterpart for $\frac{\partial T}{\partial Y}$ into (B6), we arrive at:

(B7)
$$\frac{\partial T}{\partial t}=\left[\frac{V_{x}X+V_{y}Y}{s^{2}}+\frac{V_{x}X+V_{y}Y}{2s}\rho c_{p}\sqrt{{V_{x}}^{2}+{V_{y}}^{2}}+\frac{1}{2}\rho c_{p}\left(V_{x}^{2}+V_{y}^{2}\right)\right]\frac{Q}{4\pi \sqrt{k_{x}k_{y}k_{z}}\;s}e^{-\frac{1}{2}\rho c_{p}\left(V_{x}X+V_{y}Y+\sqrt{{V_{x}}^{2}+{V_{y}}^{2}}\;s\right)}\;.$$

The term outside the brackets represents the instantaneous total temperature change ( $T-T_{\infty }$ ) at the point, as it has been shown in equation (34). By expanding the expressions within the brackets of (B7), the equation reshapes to:

(B8)
$$\frac{\partial T}{\partial t}=\frac{T-T_{\infty }}{\frac{x^{2}}{k_{x}}+\frac{y^{2}}{k_{y}}+\frac{z^{2}}{k_{z}}}\times \left[v\left(\frac{x\;\cos\;\theta }{k_{x}}+\frac{y\;\sin\;\theta }{k_{y}}\right)+\frac{1}{2}\rho c_{p}v^{2}\left(\frac{x\;\cos\;\theta }{k_{x}}+\frac{y\;\sin\;\theta }{k_{y}}\right)\sqrt{\frac{\cos^{2}\;\theta }{k_{x}}+\frac{\sin^{2}\;\theta }{k_{y}}}\;\sqrt{\frac{x^{2}}{k_{x}}+\frac{y^{2}}{k_{y}}+\frac{z^{2}}{k_{z}}}+\frac{1}{2}\rho c_{p}v^{2}\left(\frac{\cos^{2}\;\theta }{k_{x}}+\frac{\sin^{2}\;\theta }{k_{y}}\right)\left(\frac{x^{2}}{k_{x}}+\frac{y^{2}}{k_{y}}+\frac{z^{2}}{k_{z}}\right)\right].$$

A two-dimensional rotational coordinate transformation from x - y - z to $\bar{x}$ - $\bar{y}$ - $\bar{z}$ would lead to:

(B9)
$$\frac{\partial T}{\partial t}=\frac{T-T_{\infty }}{\frac{{\left(\bar{x}\cos\;\theta -\bar{y}\;\sin\;\theta \right)}^{2}}{k_{x}}+\frac{{\left(\bar{x}\;\sin\;\theta +\bar{y}\cos\;\theta \right)}^{2}}{k_{y}}+\frac{{\bar{z}}^{2}}{k_{z}}}\times \left[v\left(\frac{\left(\bar{x}\cos\;\theta -\bar{y}\;\sin\;\theta \right)\cos\;\theta }{k_{x}}+\frac{\left(\bar{x}\;\sin\;\theta +\bar{y}\cos\;\theta \right)\sin\;\theta }{k_{y}}\right)+\frac{1}{2}\rho c_{p}v^{2}\times \left(\frac{\left(\bar{x}\cos\;\theta -\bar{y}\;\sin\;\theta \right)\cos\;\theta }{k_{x}}+\frac{\left(\bar{x}\;\sin\;\theta +\bar{y}\cos\;\theta \right)\sin\;\theta }{k_{y}}\right)\times \sqrt{\frac{\cos^{2}\;\theta }{k_{x}}+\frac{\sin^{2}\;\theta }{k_{y}}}\times \sqrt{\frac{{\left(\bar{x}\cos\;\theta -\bar{y}\;\sin\;\theta \right)}^{2}}{k_{x}}+\frac{{\left(\bar{x}\;\sin\;\theta +\bar{y}\cos\;\theta \right)}^{2}}{k_{y}}+\frac{{\bar{z}}^{2}}{k_{z}}}+\frac{1}{2}\rho c_{p}v^{2}\times \left(\frac{\cos^{2}\;\theta }{k_{x}}+\frac{\sin^{2}\;\theta }{k_{y}}\right)\times \left(\frac{{\left(\bar{x}\cos\;\theta -\bar{y}\;\sin\;\theta \right)}^{2}}{k_{x}}+\frac{{\left(\bar{x}\;\sin\;\theta +\bar{y}\cos\;\theta \right)}^{2}}{k_{y}}+\frac{{\bar{z}}^{2}}{k_{z}}\right)\;\right]\;.$$

### Appendix C (equation (42) – two-dimensional solution)

The two-dimensional heat conduction equation for an orthotropic solid can be written as:

(C1)
$$k_{x}\frac{\partial^{2}T}{\partial {x^{\prime }}^{2}}+k_{y}\frac{\partial^{2}T}{\partial {y^{\prime }}^{2}}+q=\rho c_{p}\frac{\partial T}{\partial t}\;.$$

The source term q is represented by a Dirac delta function, characterizing a point heat source moving in the orthotropic plane:

(C2)
$$q=\bar{Q}\left(t\right)\delta \left(x^{\prime }-x_{0}^{\prime }\right)\delta \left(y^{\prime }-y_{0}^{\prime }\right)\;.$$

Here, $\bar{Q}$ represents the heat power per unit length, which, in a three-dimensional physical context, corresponds to the power per unit depth normal to the two-dimensional analysis plane. A transformation to a moving coordinate frame attached to the heat source is performed, along with the introduction of scaled variables (similar to equations (4), (5), (7), (8), (10), (11), (13), (14), (15), and (16) in the main text). This yields the following quasi-steady governing equation:

(C3)
$$\frac{\partial^{2}T}{\partial X^{2}}+\frac{\partial^{2}T}{\partial Y^{2}}=-\rho c_{p}V_{x}\frac{\partial T}{\partial X}-\rho c_{p}V_{y}\frac{\partial T}{\partial Y}\;.$$

Following the same approach discussed in the derivation of the three-dimensional solution (equation (19) of the main text), we introduce:

(C4)
$$T-T_{\infty }=e^{-\frac{1}{2}\rho c_{p}V_{x}X}e^{-\frac{1}{2}\rho c_{p}V_{y}Y}\varphi \left(X,Y\right)\;.$$

Substitution of equation (C4) into equation (C3) results in:

(C5)
$$\frac{\partial^{2}\varphi }{\partial X^{2}}+\frac{\partial^{2}\varphi }{\partial Y^{2}}-{\left(\frac{1}{2}\rho c_{p}\sqrt{{V_{x}}^{2}+{V_{y}}^{2}}\right)}^{2}\varphi =0\;.$$

Let us now define s as:

(C6)
$$s=\sqrt{X^{2}+Y^{2}}\;.$$

The Laplace operator in polar coordinates is:

(C7)
$$\nabla^{2}=\frac{\partial }{\partial s^{2}}+\frac{1}{s}\frac{\partial }{\partial s}+\frac{1}{s^{2}}\frac{\partial^{2}}{\partial \alpha^{2}},$$

and when is applied to φ , the angular derivative vanishes due to radial symmetry. Therefore, equation (C5) can be expressed as:

(C8)
$$\frac{\partial \varphi }{\partial s^{2}}+\frac{1}{s}\frac{\partial \varphi }{\partial s}-{\left(\frac{1}{2}\rho c_{p}\sqrt{{V_{x}}^{2}+{V_{y}}^{2}}\right)}^{2}\varphi =0\;.$$

Multiplying both sides by $s^{2}$ results in:

(C9)
$$s^{2}\frac{\partial \varphi }{\partial s^{2}}+s\frac{\partial \varphi }{\partial s}-s^{2}{\left(\frac{1}{2}\rho c_{p}\sqrt{{V_{x}}^{2}+{V_{y}}^{2}}\right)}^{2}\varphi =0\;.$$

A dimensionless variable is defined as:

(C10)
$$u=\frac{1}{2}\rho c_{p}\sqrt{{V_{x}}^{2}+{V_{y}}^{2}}\;s,$$

Substituting equation (C10) into (C9) gives:

(C11)
$$u^{2}\frac{\partial \varphi }{\partial u^{2}}+u\frac{\partial \varphi }{\partial u}-u^{2}\varphi =0\;.$$

This is the standard form of the modified Bessel differential equation of order zero. Its general solution is:

(C12)
$$\varphi =A_{1}I_{0}\left(u\right)+A_{2}K_{0}\left(u\right)\;.$$

Here, $A_{1}$ and $A_{2}$ are constants to be determined, while $I_{0}$ and $K_{0}$ are modified Bessel functions of the first and second kind, respectively, of order zero^24^:

(C13)
$$I_{0}\left(u\right)=1+\frac{{\left(\frac{1}{4}u^{2}\right)}^{1}}{{\left(1!\right)}^{2}}+\frac{{\left(\frac{1}{4}u^{2}\right)}^{2}}{{\left(2!\right)}^{2}}+\frac{{\left(\frac{1}{4}u^{2}\right)}^{3}}{{\left(3!\right)}^{2}}+\ldots ,$$

(C14)
$$K_{0}\left(u\right)=-\left[\ln\left(\frac{1}{2}u\right)+\gamma \right]I_{0}\left(u\right)+\frac{{\left(\frac{1}{4}u^{2}\right)}^{1}}{{\left(1!\right)}^{2}}+\left(1+\frac{1}{2}\right)\frac{{\left(\frac{1}{4}u^{2}\right)}^{2}}{{\left(2!\right)}^{2}}+\left(1+\frac{1}{2}+\frac{1}{3}\right)\frac{{\left(\frac{1}{4}u^{2}\right)}^{3}}{{\left(3!\right)}^{2}}+\ldots ,$$

where γ is the Euler–Mascheroni constant. As u→∞ , it is known that $I_{0}\;\to \infty$ , while $K_{0}\;\to \;0$ ^24^. Since physical constraints require the solution to decay far from the heat source (i.e., as u→∞ , then φ→ 0 ), we must set $A_{1}=0$ . Hence, the solution becomes:

(C15)
$$\varphi =A_{2}K_{0}\left(u\right)\;.$$

To evaluate $A_{2}$ , we consider the limiting case in which the velocity of the heat source v→ 0 . In this scenario, $V_{x}\to 0$ and $V_{y}\to 0$ (from equations (13) and (14) of the main text), and accordingly u→ 0 by equation (C10). Mathematically, it can be shown that:

(C16)
$$K_{0}\left(u\right)=-\ln\left(u\right)\;\text{as}\;u\to \;0^{+}\;.$$

Combining this with equation (C15) yields:

(C17)
$$\varphi =-A_{2}\;\ln\left(u\right)\;\text{as}\;v\to \;0^{+}\;.$$

Recasting equation (C2) under a transformation from the $x^{\prime }$ - $y^{\prime }$ coordinates to the X - Y coordinates yields:

(C18)
$$q=\frac{\bar{Q}}{\sqrt{k_{x}}\sqrt{k_{y}}}\;\delta \left(X\right)\delta \left(Y\right)\;.$$

At zero velocity, the steady-state heat conduction equation in the transformed coordinates with a stationary point source becomes:

(C19)
$$\frac{\partial^{2}T}{\partial X^{2}}+\frac{\partial^{2}T}{\partial Y^{2}}=-\frac{\bar{Q}}{\sqrt{k_{x}}\sqrt{k_{y}}}\;\delta \left(X\right)\delta \left(Y\right)\;.$$

We define $P=\frac{\bar{Q}}{\sqrt{k_{x}}\sqrt{k_{y}}}$ , assigning it artificial unit of [W/m]. With this definition, equation (C19) can be interpreted as steady state equation for a point heat source with power per unit of depth P located in an infinite isotropic plane with thermal conductivity of k=1 [W/m⋅K] in the X - Y coordinate system. According to Fourier’s law in radial form:

(C20)
$${\bar{q}}_{s}=-k\frac{\partial T}{\partial s}\;.$$

With attention to equation (C4) for zero velocity, it is clear that $\frac{\partial T}{\partial s}=\frac{\partial \varphi \;}{\partial s}$ . Therefore:

(C21)
$${\bar{q}}_{s}=-k\frac{\partial \varphi \;}{\partial s}=-k\frac{\partial \varphi \;}{\partial u}\frac{\partial u\;}{\partial s}=-k\left(-\frac{A_{2}}{u}\right)\left(\frac{1}{2}\rho c_{p}\sqrt{{V_{x}}^{2}+{V_{y}}^{2}}\;\right)=\frac{kA_{2}}{s}\;.$$

The radial heat flux can alternatively be obtained by dividing the input heat power per unit depth by the perimeter of a circular contour centered at the point heat source. Accordingly, the following relation must hold:

(C22)
$$\frac{P}{2\pi s}=\frac{kA_{2}}{s}\;.$$

With attention to the definition of P , solving for $A_{2}$ yields:

(C23)
$$A_{2}=\frac{\bar{Q}}{2\pi \sqrt{k_{x}k_{y}}\;}\;.$$

Now, for a general case (i.e., arbitrary velocity v ), substituting equations (C10) and (C23) into the general solution (C15), and then applying equation (C4), results in:

(C24)
$$T=T_{\infty }+\frac{\bar{Q}}{2\pi \sqrt{k_{x}k_{y}}\;}e^{-\frac{1}{2}\rho c_{p}\left(V_{x}X+V_{y}Y\right)}K_{0}\left(\frac{1}{2}\rho c_{p}\sqrt{{V_{x}}^{2}+{V_{y}}^{2}}s\right)\;.$$

Rewriting in terms of the x - y coordinates:

(C25)
$$T=T_{\infty }+\frac{\bar{Q}}{2\pi \sqrt{k_{x}k_{y}}\;}e^{-\frac{1}{2}\rho c_{p}v\left(\frac{x\;\cos\;\theta }{k_{x}}+\frac{y\;\sin\;\theta }{k_{y}}\;\right)}K_{0}\left(\frac{1}{2}\rho c_{p}v\;\sqrt{\frac{\cos^{2}\;\theta }{k_{x}}+\frac{\sin^{2}\;\theta }{k_{y}}}\;\sqrt{\frac{x^{2}}{k_{x}}+\frac{y^{2}}{k_{y}}}\right)\;.$$

A final coordinate transformation from the x - y (i.e., oriented along the principal material axes) to a rotated system of $\bar{x}$ - $\bar{y}$ , where $\bar{x}$ aligns with the direction of source motion, results in:

(C26)
$$T=T_{\infty }+\frac{\bar{Q}}{2\pi \sqrt{k_{x}k_{y}}\;}\left[e^{-\frac{1}{2}\rho c_{p}v\left(\frac{\left(\bar{x}\cos\;\theta -\bar{y}\;\sin\;\theta \right)\cos\;\theta }{k_{x}}+\frac{\left(\bar{x}\;\sin\;\theta +\bar{y}\cos\;\theta \right)\sin\;\theta }{k_{y}}\;\right)}\right]\times K_{0}\left(\frac{1}{2}\rho c_{p}v\;\sqrt{\frac{\cos^{2}\;\theta }{k_{x}}+\frac{\sin^{2}\;\theta }{k_{y}}}\;\sqrt{\frac{{\left(\bar{x}\cos\;\theta -\bar{y}\;\sin\;\theta \right)}^{2}}{k_{x}}+\frac{{\left(\bar{x}\;\sin\;\theta +\bar{y}\cos\;\theta \right)}^{2}}{k_{y}}}\right).$$

The validity of equation (C26) was assessed by comparing its predictions with two-dimensional finite element simulations conducted using COMSOL Multiphysics. The computational domain consisted of an orthotropic rectangular plate, with a point heat source placed at the center of the rectangle. The mesh employed 277,458 triangular elements, with a minimum element size of 2 × $10^{-7}$ m near the heat source and a mesh growth rate of 1.03 toward the domain boundaries. The parameters $k_{x}$ , $k_{y}$ , ρ , $c_{p}$ , v , and $T_{\infty }$ were all set according to the values provided in Table 1. The heat source intensity $\bar{Q}$ was specified as 2000 W/m. Figure C1 shows the resulting isothermal contours, ranging from T = 60°C to T = 220°C in increments of 20°C, for three different orientations of the material principal axes relative to the heat source velocity, namely θ = 0°, 45°, and 90°, displayed from top to bottom, respectively.
